# Wnt5A regulates ABCB1 expression in multidrug-resistant cancer cells through activation of the non-canonical PKA/β-catenin pathway

**DOI:** 10.18632/oncotarget.2631

**Published:** 2014-10-24

**Authors:** Tsai-Hsien Hung, Sheng-Chi Hsu, Ching-Yi Cheng, Kong-Bung Choo, Ching-Ping Tseng, Tse-Ching Chen, Ying-Wei Lan, Tsung-Teng Huang, Hsin-Chih Lai, Chuan-Mu Chen, Kowit-Yu Chong

**Affiliations:** ^1^ Graduate Institute of Biomedical Sciences, Division of Biotechnology, College of Medicine, Chang Gung University, Tao-Yuan, Taiwan, Republic of China; ^2^ Department of Medical Biotechnology and Laboratory Science, College of Medicine, Chang Gung University, Tao-Yuan, Taiwan, Republic of China; ^3^ Molecular Medicine Research Center, College of Medicine, Chang Gung University, Tao-Yuan, Taiwan, Republic of China; ^4^ Cancer Molecular Diagnostic Laboratory, Chang Gung Memorial Hospital, Lin-Kou Medical Center, Tao-Yuan, Taiwan, Republic of China; ^5^ Department of Pathology, Chang Gung Memorial Hospital, Lin-Kou Medical Center, Tao-Yuan, Taiwan, Republic of China; ^6^ Department of Cosmetic Science, Graduate Institute of Health Industry Technology, Research Center for Industry of Human Ecology, Chang Gung University of Science and Technology, Tao-Yuan, Taiwan, Republic of China; ^7^ Department of Preclinical Sciences, Faculty of Medicine and Health Sciences and Centre for Stem Cell Research, Universiti Tunku Abdul Rahman, Selangor, Malaysia; ^8^ Center for Molecular and Clinical Immunology, College of Medicine, Chang Gung University, Tao-Yuan, Taiwan, Republic of China; ^9^ Department of Life Sciences, National Chung Hsing University, Taichung, Taiwan, Republic of China; ^10^ Agricultural Biotechnology Center, National Chung Hsing University, Taichung, Taiwan, Republic of China; ^11^ Rong-Hsing Translational Medicine Center, National Chung Hsing University, Taichung, Taiwan, Republic of China

**Keywords:** Multiple Drug Resistance, Wnt5A, shRNA, Cell cycle, apoptosis

## Abstract

Multidrug resistance in cancer cells arises from altered drug permeability of the cell. We previously reported activation of the Wnt pathway in ABCB1-overexpressed human uterus sarcoma drug-resistant MES-SA/Dx5 cells through active β-catenin and associated transactivation activities, and upregulation of Wnt-targeting genes. In this study, Wnt5A was found to be significantly upregulated in MES-SA/Dx5 and MCF7/ADR2 cells, suggesting an important role for the Wnt5A signaling pathway in cancer drug resistance. Higher cAMP response elements and Tcf/Lef transcription activities were shown in the drug-resistant cancer cells. However, expression of Wnt target genes and CRE activities was downregulated in Wnt5A shRNA stably-transfected MES-SA/Dx5 cells. Cell viability of the drug-resistant cancer cells was also reduced by doxorubicin treatment and Wnt5A shRNA transfection, or by Wnt5A depletion. The *in vitro* data were supported by immunohistochemical analysis of 24 paired breast cancer biopsies obtained pre- and post-chemotherapeutic treatment. Wnt5A, VEGF and/or ABCB1 were significantly overexpressed after treatment, consistent with clinical chemoresistance. Taken together, the Wnt5A signaling pathway was shown to contribute to regulating the drug-resistance protein ABCB1 and β-catenin-related genes in antagonizing the toxic effects of doxorubicin in the MDR cell lines and in clinical breast cancer samples.

## INTRODUCTION

Multidrug resistance (MDR) in cancer is a condition in which cancer cells become resistant to structurally unrelated anticancer drugs [1]. Several mechanisms have been reported to activate MDR in such cancers, including reduced apoptosis, advanced DNA damage repair mechanisms, altered drug metabolism, mutation or over expression of the drug's targets, drug inactivation, or efflux of the drug from the cell [2]. P-glycoprotein 1 (permeability glycoprotein, abbreviated as P-gp or Pgp), also known as multidrug resistance protein 1 (MDR1) or ATP-binding cassette sub-family B member 1 (ABCB1) or cluster of differentiation 243 (CD243), is a glycoprotein that, in humans, is encoded by the ABCB1 gene. Overexpression of ABCB1 causes therapeutic drugs to be pumped out of the cancer cells thus preventing effective treatment. Since ABCB1 expression in normal tissues leads to higher toxicity in cancer treatment using an ABCB1 inhibitor [3], researchers have focused on the molecular pathway of MDR cancer cells as a strategy in the development of new molecular targets [4].

Wnt proteins are crucial signaling molecules which are involved not only in physiological development [5], decision of stem cell fate [6] but also in oncogenesis [7] and cross-talk between signaling pathways [8, 9]. Canonical Wnt proteins such as Wnt3A stabilize β-catenin in the nucleus through binding with the Frizzle proteins of the cell membrane [10]. Furthermore, Wnt5A was a well-discussed non-canonical Wnt protein that plays an important role in many physiological processes, including planar cell polarity (PCP) [11], Wnt calcium signaling pathway [12], axon guidance [13], stem cell functions [14], antagonization of canonical Wnt proteins [15], and macrophage-induced malignant invasion [16]. Although Wnt-5A is thought to have primarily functions in the non-canonical pathway, it also has functions in β-catenin stabilization [17].

Recent studies indicated that ABCB1 expression was controlled by Wnt signaling in MDR cancer cells [18]. Furthermore, Wnt signaling inhibition resensitizes MDR cancer cells to chemotherapy drugs [19]. However, these studies have been confined to canonical Wnt protein-related signaling of drug resistant cancer cells. Furthermore, our previous studies indicated that Wnt-targeting genes, including c-Myc and cyclin D1, were upregulated and were relevant in inhibiting the expression of p21 in MES-SA/Dx5 cells. Moreover, we also found that the canonical Wnt pathway was only activated in ABCB1-overexpressed MES-SA/Dx5 cells through active β-catenin and related transactivation activities regulating the cell cycle [20].

Since little is known about the non-canonical Wnt protein-related pathway in drug resistant cancer cells, we have attempted in this study to investigate the role of the non-canonical Wnt5A in drug-resistant cancer cells. Herein, the non-canonical Wnt5A was upregulated in two multidrug-resistant cancer cell lines, MES-SA/Dx5 and MCF7/ADR2 cells. The data indicated that Wnt5A stabilized β-catenin in the nucleus through the PKA pathway in both drug-resistant cells. Furthermore, neutralization of Wnt5A inhibited growth of the drug-resistant cells. Moreover, inhibition of Wnt5A by shRNA led to resensitization of the cells to doxorubicin *in vitro* and *in vivo*. Besides, Wnt5A expression level was positively correlated with ABCB1 and VEGF expression levels in the clinical breast cancer tissues. Taken together, Wnt5A-controlled PKA/β-catenin signaling regulate upregulation of ABCB1, cyclin D1, c-Myc and VEGF in MDR cancer cells for increased survival advantage under chemotherapeutic treatment.

## RESULTS

### Wnt signaling is activated in two multidrug-resistant cancer cell lines

**Figure 1:**
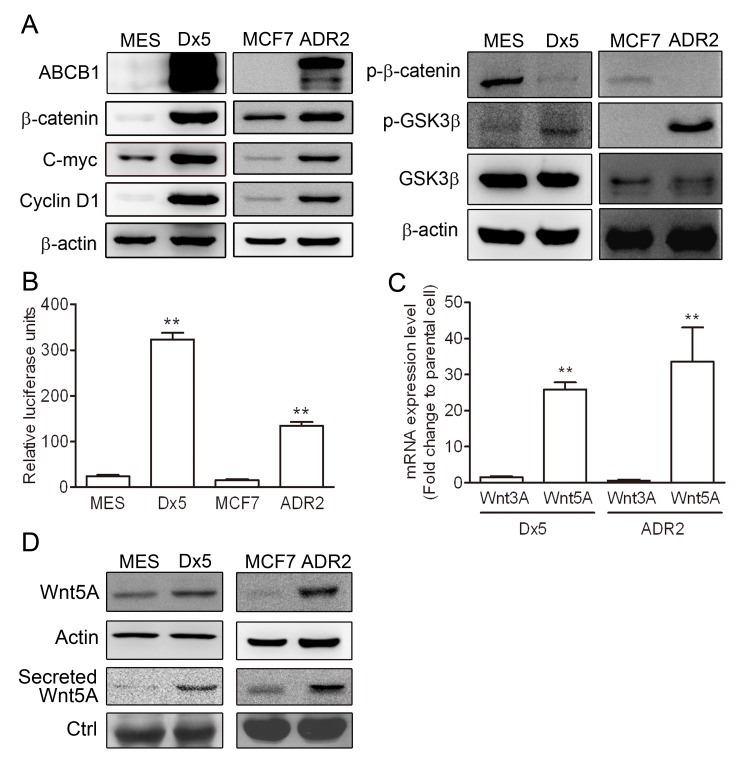
The Wnt pathway is upregulated in drug-resistant cancer cells (A) Western blot analysis of ABCB1, β-catenin, phosphorylated β-catenin, GSK3β, phosphorylated GSK3β, c-Myc and cyclin D1 protein expression levels in MES-SA, MES-SA/Dx5, MCF-7 and MCF-7/ADR2 cells. (B) β-catenin transactivation activity assayed by TOP assay in both MDR cells. (C) Quantitative real-time PCR assays of Wnt3A and Wnt5A mRNA expression levels in two MDR cells. (D) Western blot analysis of Wnt5A protein levels in the two MDR cancer cells. Data represent mean ± S.D. of three independent experiments. *P<0.05 and **P<0.01 indicate differences between the drug-resistant and the parental cells.

Since the Wnt pathway is correlated to ABCB1 expression level in multidrug resistant cells [18], the ABCB1, β-catenin, c-Myc and cyclin D1 protein levels were evaluated in the parental uterus sarcoma MES-SA and the breast cancer MCF7 cell lines in relation to their drug resistant counterparts, MES-SA/Dx5 and MCF7/ADR2, respectively. The data showed higher expression levels of ABCB1, β-catenin, c-Myc and cyclin D1 in the drug-resistant MES-SA/Dx5 and MCF7/ADR2 cells (Figure 1A). Furthermore, lower phosphorylation status of β-catenin (Thr41/Ser45) and higher phosphorylation levels of GSK3β (Ser9) were also detected in MES-SA/Dx5 and MCF7/ADR2 cells (Figure 1A). Expression levels of the Wnt genes were verified by quantitative RT-PCR. The data indicated that Wnt2, Wnt5A and Wnt7A were upregulated in MES-SA/Dx5 cells compared to MES-SA cells (Supplementary Figure 1A). Interestingly, Wnt5A alone, among other Wnt genes, showed significantly increased levels in MES-SA/Dx5 (Supplementary Figure 1B). In both cases, elevated TOP activities and higher Wnt5A mRNA levels were concurrently detected in MES-SA/Dx5 and MCF7/ADR2 cells (Figures 1B and C). The data indicated that Wnt/β-catenin and Wnt5A were upregulated in MDR cells. Wnt5A expression level was assessed by western blot, which demonstrated that higher expression levels of cellular Wnt5A and secreted Wnt5A in MES-SA/Dx5 and MCF7/ADR2 cells (Figure 1D). Furthermore, higher expression levels of cellular Wnt5A and secreted Wnt5A were also observed in the multidrug-resistant KB-V1, NCI/ADR-RES and SW620-MDR1 cells of the cervix, ovary and colon, respectively (Supplementary Figure 2). These data indicated that elevated expression of Wnt5A regulated the signaling pathway to support MDR cancer cell resistant to chemotherapy.

### Upregulation of Wnt5A was controlled by DNA methylation in drug-resistant cell line

**Figure 2:**
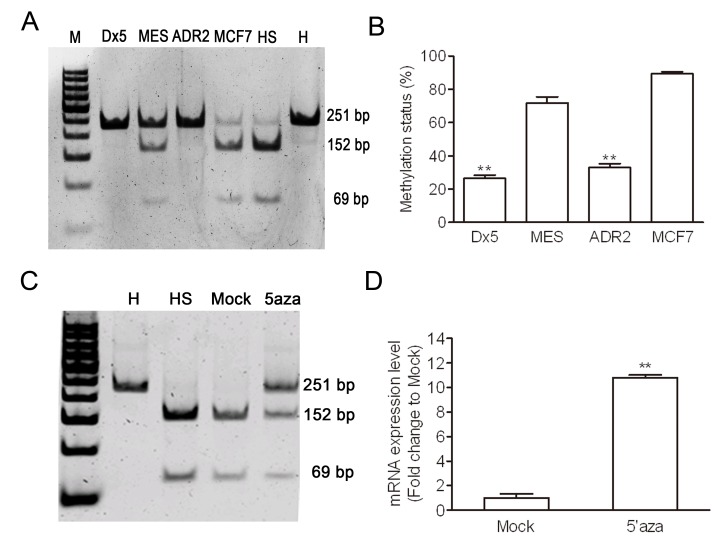
Hypomethylation of Wnt5A in drug-resistant cancer cell lines (A) Methylation status was detected by COBRA assay in both MDR cells. (B) Percentages of digested band intensity of the gel in cell lines were quantitated by NIH ImageJ software. Data represent mean ± S.D. of three independent experiments. **P<0.01 indicates differences between the drug-resistant and the parental cells. (C) Methylation status was detected by COBRA assay in 48 hours 5-aza-cytidine treated MES-SA cells. (D) The Wnt5A expression in MES-SA cell with 5-aza-cytidine 48 hours treatment were detected by quantitative realtime PCR assay. H represents a human leukocyte DNA control sample. HS represents M.SssІ-treated leukocyte DNA. Data represent mean ± S.D. of three independent experiments. **P<0.01 indicates differences between the Mock treated 5-aza-cytidine treated MES-SA cells.

In order to determine whether there was aberrant methylation of Wnt5A, we used COBRA assay to further investigate the CpG island of the first intron of Wnt5A (+494-+664) in MES-SA/Dx5 and MCF7/ADR2 cells. The COBRA assay results indicated lower methylation status in MES-SA/Dx5 and MCF7/ADR2 cells (Figure 2A and 2B). To determine whether expression of Wnt5A in MES-SA/Dx5 cells is regulated by DNA methylation of the CpG island in the first intron of Wnt5A (+494-+664), Wnt5A expression was examined in untreated and 2 μM 5-aza-2′-deoxycytidine–treated MES-SA cells. 5-aza-2′-deoxycytidine was previously reported to inhibit DNMT1 and reduced DNA hypomethylation in a dose-dependent manner [21]. Quantitative RT-PCR data indicated that 5-aza-2′-deoxycytidine-treated MES-SA cells, which had lower methylation status, showed 11-fold increase of Wnt5A mRNA expression level (Figure 2C and 2D). These data indicated that aberrant hypomethylation of the Wnt5A intron led to elevated Wnt5A expression levels in MDR cancer cells.

### Cyclic AMP-dependent protein kinase A pathway is upregulated in drug-resistant cells

**Figure 3:**
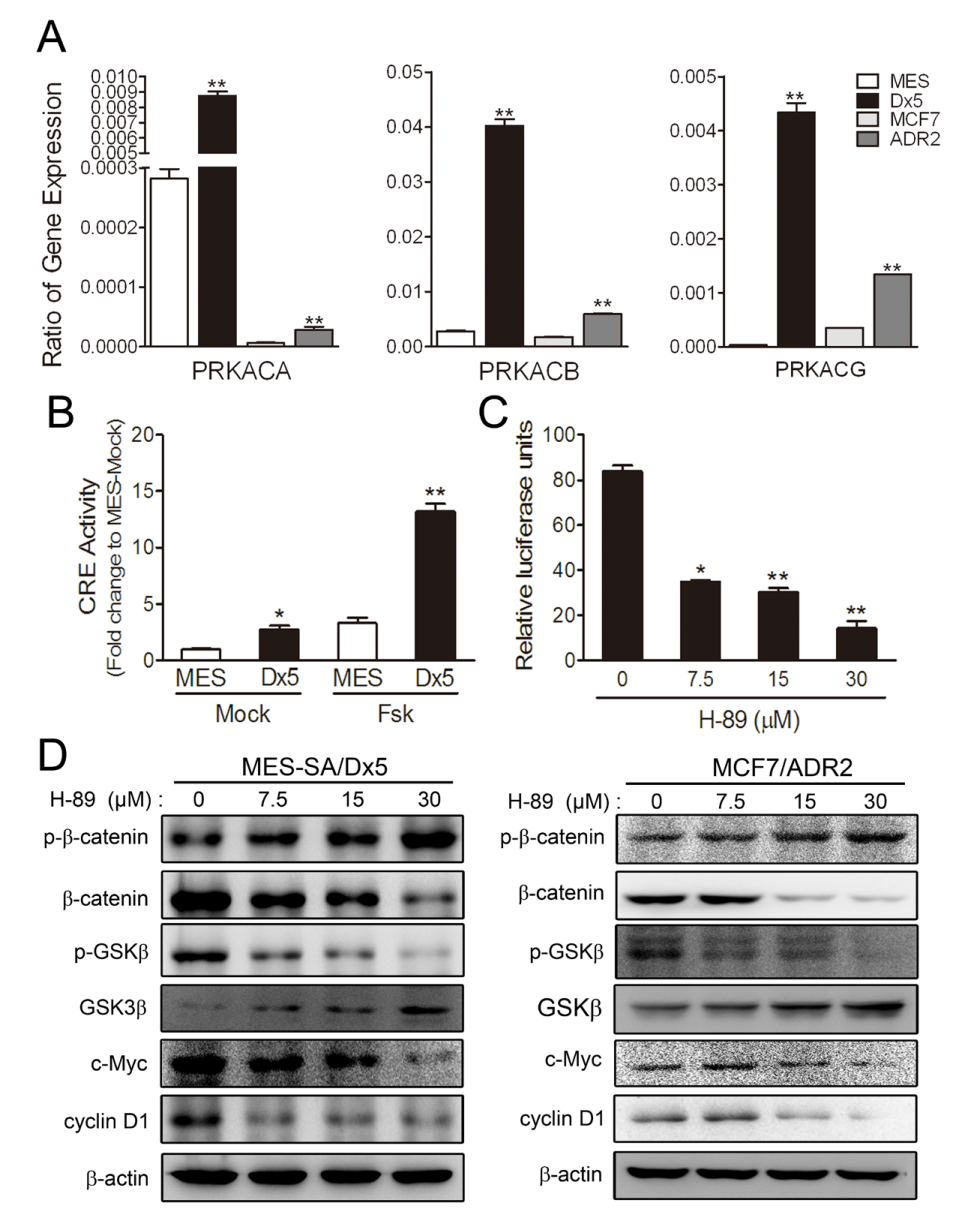
Cyclic AMP-dependent protein kinase A pathway is upregulated in two drug-resistant cancer cell lines (A) Real-time RT-PCR analysis of PRKACA, PRKACB and PRKACG mRNA expression in MES-SA, MES-SA/Dx5, MCF7 and MCF7/ADR2 cells. (B) Activities of CRE pathway of 10 μM foskolin-treated MES-SA and MES-SA/Dx5 cells. *P<0.05 and **P<0.01 indicate differences between the drug-resistant and the parental cells. (C) Effects of protein kinase A inhibitor, H-89 on TOP reporter-transfected MES-SA/Dx5 cells. *P*<0.05 and **P<0.01 indicate differences between the mock and the H-89 treated MES-SA/Dx5 cells. (D) Effects of H-89 on β-catenin, phosphorylated β-catenin, GSK3, phosphorylated GSK3β, c-Myc and cyclin D1 protein expression levels in MES-SA/Dx5 and MCF7/ADR2 at the indicated concentration after 48 hours of treatment as analyzed in western blots. Data represent mean ± S.D. of three independent experiments.

Higher Wnt5A and β-catenin expression levels were observed in drug-resistant MES-SA/Dx5 and MCF7/ADR2 cells (Figure 1A and 1D). A previous report has indicated that Wnt5A-activated Protein Kinase A pathway leads to β-catenin accumulation in the nucleus via GSK3β inactivation [22]. To verify possible Wnt5A pathway activation resulting in β-catenin induction, we evaluated the expression of cAMP-dependent protein kinase catalytic subunit PRKACA, PRKACB and PRKACG in MES-SA/Dx5 and MCF7/ADR2 cells. Quantitative RT-PCR data showed that PRKACB expression was significantly increased in MES-SA/Dx5 and MCF7/ADR2 cells (Figure 3A). It has previously been shown that PKA is activated by increasing the transcription of its catalytic subunit β, PRKACB [23]. These data led us to hypothesize upregulation of cAMP/PKA activities in drug-resistant cells. Previous data indicated that PKA activation was followed by phosphorylation of cyclic AMP response element (CRE)-binding proteins and subsequent CRE activation, leading to expression of CRE-regulated genes [24]. To evaluate the PKA activity, CRE reporter assays were performed and the data indicated that CRE expressed was upregulated in MES-SA/Dx5 and foskolin-treated MES-SA/Dx5 cells (Figure 3B). Furthermore, higher cAMP levels were detected in MES-SA/Dx5 cells (Supplementary Figure 3). After treating with increasing dosages of the PKA inhibitor H-89 in MES-SA/Dx5 and MCF7/ADR2, TOP reporter assays showed decreased activities (Figure 3C) and western blot analysis showed increases of phosphorylated β-catenin (Thr41/Ser45) and GSK3β in a dose-dependent manner (Figure 3D). On the other hand, decreased levels of β-catenin, phosphorylated GSK3β (Ser 9), c-Myc and cyclin D1 were also found in H-89-treated MES-SA/Dx5 and MCF7/ADR2 cells in a dose-dependent manner (Figure 3D). These data indicated that upregulating PKA signaling was detected and the β-catenin-related signaling was regulated by PKA signaling in MDR cells.

### Wnt5A regulates PKA and β-catenin activities in the drug-resistant MES-SA/Dx5 cell line

**Figure 4:**
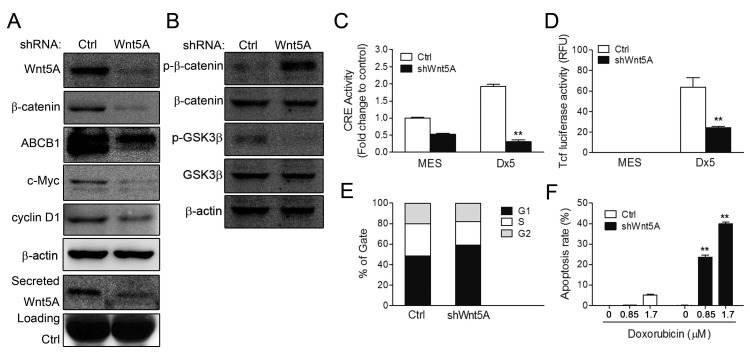
CRE and β-catenin activities and cell viability are reduced in Wnt5A shRNA-knockdown MES-SA/Dx5 cell line (A-B) Western bot analysis of expression levels of (A) Wnt5A, β-catenin, c-Myc and cyclin D1 and secreted Wnt5A expression levels and (B) β-catenin, phosphorylated β-catenin, GSk3,.phosphorylated GSk3β in shWnt5A transient transfected MES-SA/Dx5 cells. (C) CRE activities and (D) β-catenin transactivation activities were detected in shWnt5A transient transfected MES-SA and MES-SA/Dx5 cells. (E) Cell cycle assay by PI staining in shWnt5A transient transfected MES-SA/Dx5 cells. (F) Apoptosis rate in doxorubicin-treated Wnt5A-knockdown stably-transfected cells. Data represent mean ± S.D. of three independent experiments. *P<0.05 and **P<0.01 indicate differences between the shWnt5A and control shRNA transfected cells.

To confirm the regulatory role of Wnt5A on β-catenin through PKA activity, a Wnt5A shRNA vector was introduced into MES-SA/Dx5 cells. Western blot data indicated that β-catenin, ABCB1, c-Myc and cyclin D1 were downregulated in Wnt5A-knockdown MES-SA/Dx5 cells (Figure 4A). Likewise, Wnt5A secretion was also decreased in these cells (Figure 4A). Besides, increases in phosphorylated β-catenin (Thr41/Ser45) and decreased levels of phosphorylated GSK3β (Ser 9) and β-catenin were also detected Wnt5A-knockdown MES-SA/Dx5 cells (Figure 4B). Furthermore, CRE and TOP reporter assays indicated that CRE and TOP activities were reduced in MES-SA/Dx5 cells when Wnt5A was knocked down (Figures 4C and 4D). Since decreased cyclin D1 levels were detected, PI staining was employed to examine cell cycle changes in Wnt5A-knockdown MES-SA/Dx5 cells, which showed that cells in G1 phase increased 50% to 60% in the Wnt5A knockdown MES-SA/Dx5 cells relative to control vector transfected MES-SA/Dx5 cells (Figure 4E). Moreover, TUNEL assays showed that doxorubicin-treated Wnt5A-knockdown MES-SA/Dx5 cells were set in the course of apoptosis, and FACS analysis further showed that the apoptotic rate was 0% and 5% in 0.85 μM and 1.7 μM doxorubicin-treated control knockdown MES-SA/Dx5 cells, but apoptosis reached up to 25% and 43% in similarly-treated Wnt5A-knockdown MES-SA/Dx5 cells (Figure 4F). These data indicated that Wnt5A controlled PKA and β-catenin signaling in MDR cells.

### ABCB1 expression and efflux ability are inhibited in the Wnt5A-knockdown drug-resistant MES-SA/Dx5 cell line

**Figure 5:**
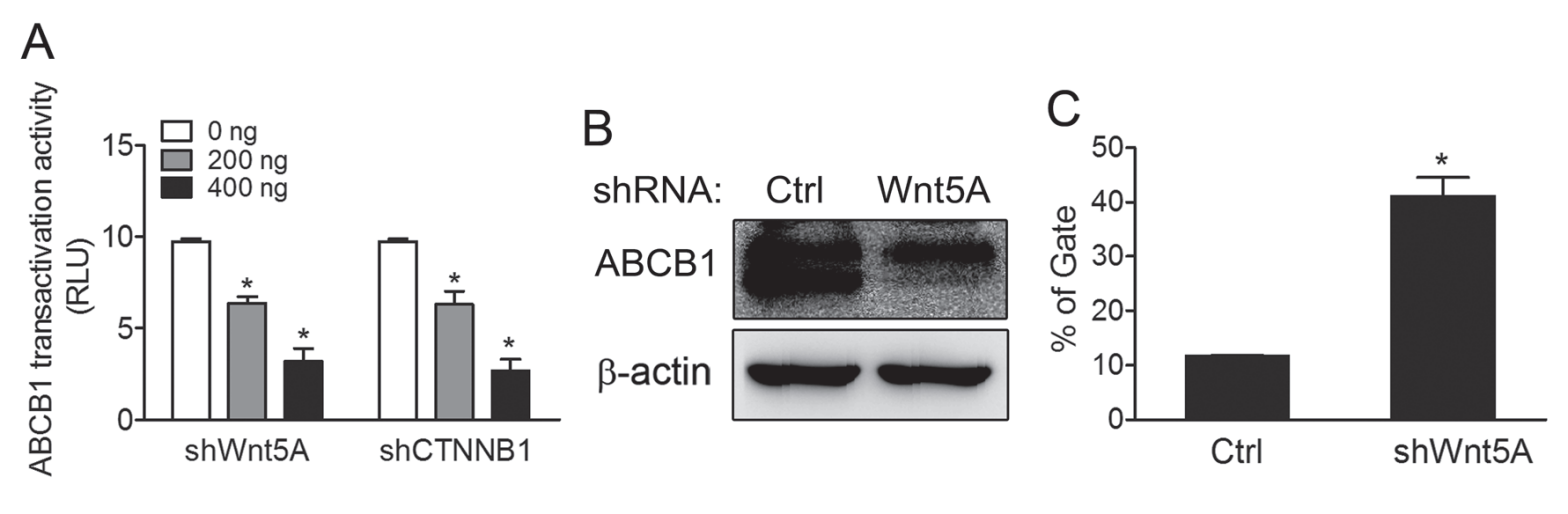
ABCB1 pump-out ability is reduced in Wnt5A shRNA- knockdown MES-SA/Dx5 cells (A) ABCB1 transactivation activities in ABCB1 promoter-luciferase reporter assays in shWnt5A and shCTNNB1-transfected MES-SA/Dx5 cells. (B) Western blot analysis of ABCB1 expression levels and (C) Pump out abilities by Calcein AM staining in shWnt5A-transfected MES-SA/Dx5 cells. Data represent mean ± S.D. of three independent experiments. *P<0.05 and **P<0.01 indicate differences between the shWnt5A and control shRNA transfected cells.

Transcriptional control of ABCB1 by the β-catenin/TCF response elements in the promoter has previously been reported [18]. As data presented in the preceding sections showed that β-catenin was regulated by Wnt5A in drug-resistant cells, we therefore further evaluated expression levels and functions of ABCB1 in Wnt5A-knockdown MES-SA/Dx5 cells. Data from ABCB1-luciferase reporter assays, quantitative RT-PCR and western blot analysis showed that ABCB1 promoter activities and protein expression levels were reduced in Wnt5A-knockdown MES-SA/Dx5 cells (Figure 5A and B). Calcein AM is a useful dye for quantitative functional analysis of ABCB1; lower fluorescence in Calcein AM-stained cells means higher drug pump-out ability [25]. When the dye was used to monitor the ABCB1 functions in Wnt5A-knockdown MES-SA/Dx5 cells, the data showed that the percentage of Calcein AM-stained cell was increased by 40% in the Wnt5A knockdown cells compared with the control cells (Figure 5C).

### Reduction of tumor volume in Wnt5A shRNA-knockdown MES-SA/Dx5 cells in vivo

**Figure 6:**
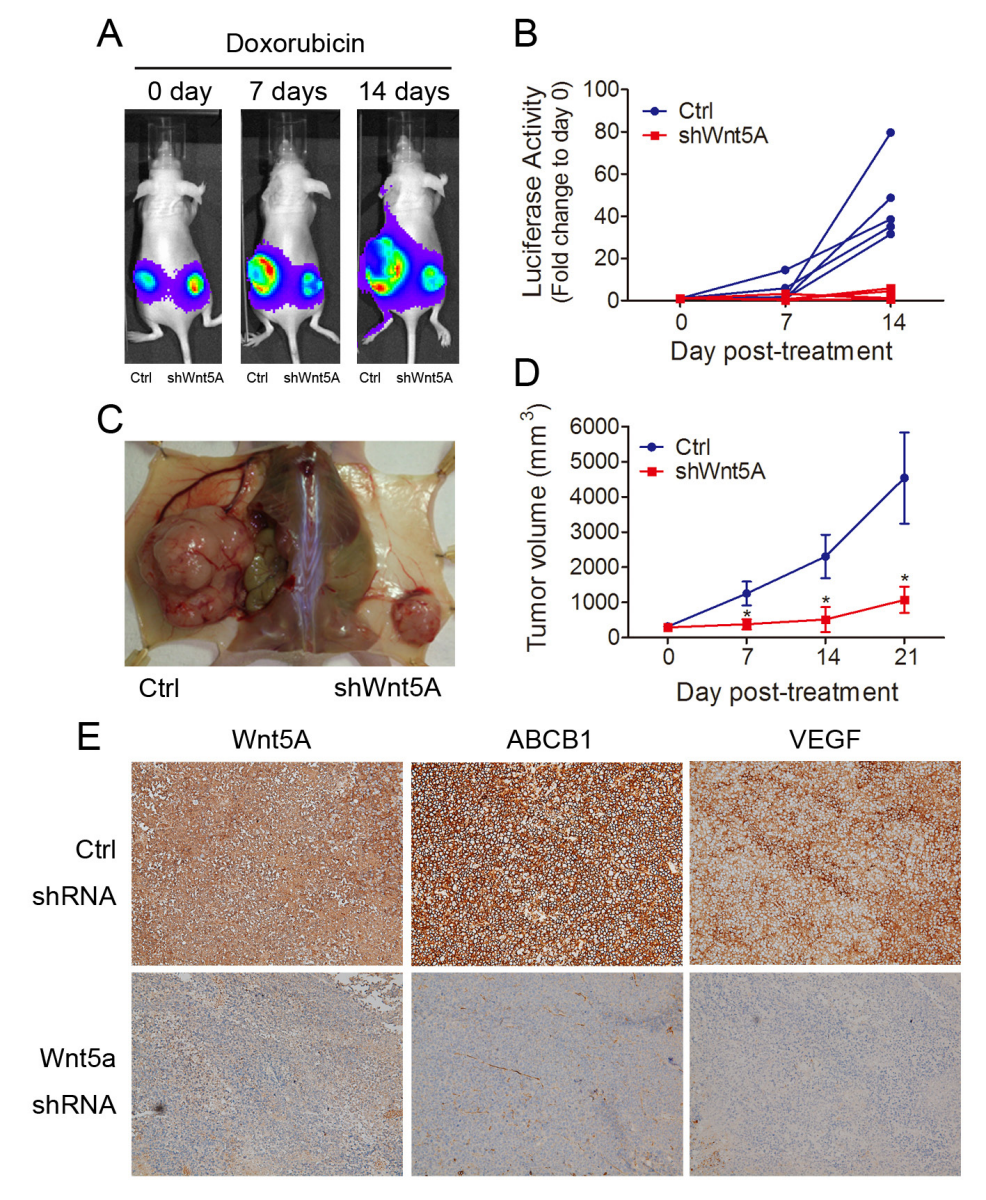
*In vivo* reduction of tumor growth rate of doxorubicin-treated Wnt5A shRNA-knockdown MES-SA/Dx5 cells (A) Tumor formation of biweekly doxorubicin-treated shWnt5A (right) and control (left) vector-transfected MES-SA/Dx5 cells that were subcutaneously transplanted into nude mice. Tumor formation was monitored by using Bioluminescence imaging at day 0, 7 and 14 after doxorubicin treatment. (B)Tumor burden monitored by Bioluminescence imaging of shWnt5A (red) and control shRNA (blue) cell-transplanted nude mice at the indicated time points after doxorubicin treatment. (n=5). (C) Images of tumor of shWnt5A (right) and control shRNA cell-transplanted (left) nude mice at day 21 after treatment with doxorubicin. (D) tumor volume of shWnt5A (red) and control shRNA (blue) cell-transplanted nude mice at the indicated time points after doxorubicin treatment. (n=5). (E) Immunohistochemical staining of Wnt5A, ABCB1 and VEGF in Wnt5A shRNA- and control scramble shRNA-transduced MES-SA/Dx5 cells. *P<0.05 and **P<0.01 indicate differences between xenograft of the shWnt5A and control shRNA transfected cells.

As shown above, Wnt5A knockdown improved doxorubicin sensitivity of MES-SA/Dx5 cells *in vitro*. To evaluate these observations *in vivo,* the tumor size and viability of doxorubicin-treated Wnt5A-knockdown MES-SA/Dx5 xenografts were determined. The IVIS luciferase imaging results indicated that 7 or 14 days of doxorubicin treatment led to slower growth in Wnt5A-knockdown tumors when compared with the xenografts of the vector control (Figure 6A and B). When tumors were collected after 21 days of doxorubicin treatment, reduced tumor sizes from the Wnt5A-knockdown groups were observed (Figure. 6C). The tumor sizes of xenografts of Wnt5A-knockdown MES-SA/Dx5 cells were 2-, 3- and 4-fold reduced in the mean relative tumor volume compared to those of the vector control cells at 7, 14 and 21 days of doxorubicin treatment, respectively (Figure. 6D). Furthermore, xenografts of Wnt5A-knockdown MES-SA/Dx5 cells showed reduced expression levels of ABCB1 and VEGF (Figure. 6E). The data demonstrated tumor regression in the Wnt5A-knockdown tumor treated with doxorubicin, suggesting that this Wnt5A-related pathway contributes to MDR tumor progression.

### Antibody neutralization of Wnt5A leads to inhibition of β-catenin-related pathway and reduces cell viability in drug-resistant cells

**Figure 7:**
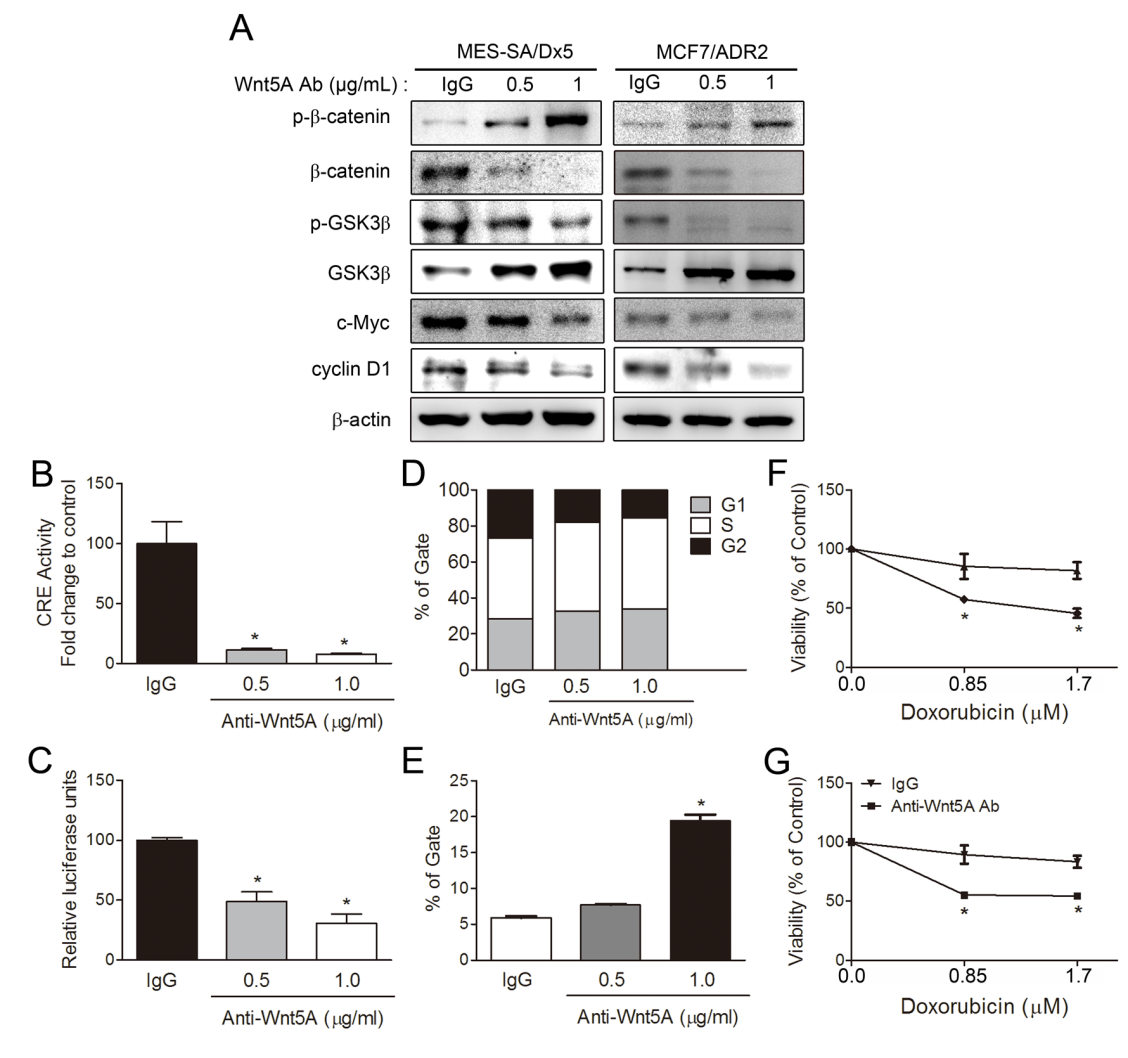
Antibody depletion of Wnt5A leads to downregulation of PKA/β-catenin activities and resensitizes doxorubicin toxicity in MES-SA/Dx5 cells (A) Western blot analysis of β-catenin, phosphorylated β-catenin, GSK3β, phosphorylated GSK3β, c-Myc, and cyclin D1 protein expression levels in Wnt5A antibody-treated MES-SA/Dx5 and MCF7/ADR2 cells. PKA and β-catenin activities as monitored by (B) CRE and (C) TOP reporter assays, (D) PI staining in cell cycle analysis and (E) Calcein AM assay of drug pump out ability in Wnt5A antibody treated-MES-SA/Dx5 cell. Values are expressed as the mean ± S.D. (n=3). *P<0.05 and **P<0.01 indicate differences between the antibody and IgG treated-cells. MTT assays of cell viability in doxorubicin and Wnt5A antibody-cotreated (F) MCF7/ADR2 cells and (G) MES-SA/Dx5. Values are expressed as the mean ± S.D. (n=3). *P<0.05 and **P<0.01 indicate differences between the drug-resistant and the parental cells.

As Wnt5A is a secreted protein that affects cells in an autocrine or paracrine manner, activation of Wnt5A/PKA/β-catenin pathway was further confirmed in drug-resistant cancer cells by depleting Wnt5A using a neutralizing anti-Wnt5A antibody. Western blot analysis showed that β-catenin, c-Myc and cyclin D1 of MES-SA/Dx5 and MCF7/ADR2 were decreased in a dose-dependent manner in anti-Wnt5A antibody treatment (Figure 7A). Increased levels of GSK3β were also observed in anti-Wnt5A antibody treated-MES-SA/Dx5 and MCF7/ADR2 cells (Figure 7A). On the other hand, the data revealed that CRE and TOP activities of MES-SA/Dx5 were significantly reduced with anti-Wnt5A antibody in a dose-dependent manner (Figure 7B and 7C). Since lower levels of cyclin D1 were detected, cell cycle progression was further assessed in the anti-Wnt5A antibody-treated MES-SA/Dx5 cells, which resulted in a delay in cell cycle progression through G1/S and S/G2. In control antibody-treated MES-SA/Dx5 cells, 28.73% of cells were in the G1 phase and 40.61% of cells were in the S phase. In the presence of 0.5 or 1 μg anti-Wnt5A antibody, 32.74% and 33.61% of cells were in the G1 phase, while 49.34% and 50.87% of cells remained in S phase, respectively (Figure 7D). As decreased β-catenin expression levels were observed in anti-Wnt5A antibody-treated MES-SA/Dx5 cells, and β-catenin/Tcf is an important transcriptional regulator of ABCB1, the drug efflux ability was next evaluated in the anti-Wnt5A antibody-treated MES-SA/Dx5 cells. The data showed that the mean fraction of calcein AM-stained cells was 5.9% in the control antibody-treated MES-SA/Dx5 cells, but cell fraction was increased to 7.6% and 19.4% when 0.5 or 1 μg anti-Wnt5A antibody was applied (Figure 7E). Moreover, MTT assay data revealed that the cell viability was 89.5% and 85.4% in 1 μg control antibody and 0.85 μM doxorubicin co-treated MCF7/ADR2 and MES-SA/Dx5 cells, respectively. Specifically, after a combined application of 1 μg anti-Wnt5A antibody with 0.85 μM doxorubicin, a further reduction in cell viability of 55.3% and 57.4% was observed in MCF7/ADR2 and MES-SA/Dx5, respectively (Figure 7F and 7G). The data demonstrated that the drug pump out ability and cell viability were decreased in Wnt-5A-depleted MCF7/ADR2 and MES-SA/Dx5 cells, further supporting the secretion of Wnt5A to control the PKA/β-catenin pathway in MDR cells.

### Expression of Wnt5A, ABCB1 and VEGF is associated with clinical chemoresistance in breast cancer patients

**Figure 8:**
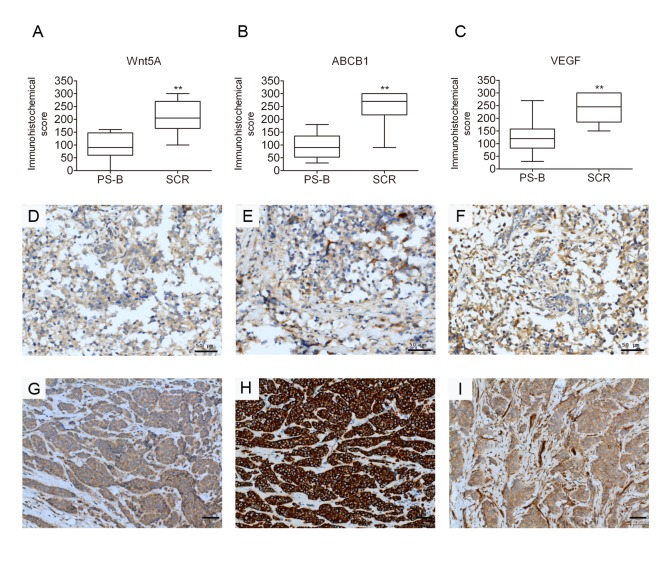
Expression of Wnt5A, ABCB1 and VEGF is associated with clinical chemoresistance in breast cancer patients Immunohistochemical analysis of expression of (A) Wnt5A, (B) ABCB1 and (C) VEGF in a clinical cohort of patient matched-tumour specimens from PS-B, primary surgery, breast (naive to chemotherapy) and SCR, secondary surgery (after tumour has recurred and after chemotherapy) breast cancer tumor biopsies of relapsed patients. (D-I) Analysis of breast cancer tissue biopsies for expression of Wnt5A (D,G), ABCB1 (E,H) and VEGF(F,I) in primary and relapsed tumor samples. Expression in Immunohistochemical scores are scored by multiplying the percentage of positive cells (P) by the intensity (I). Formula: Q = P × I; Maximum = 300. Scale bar: 50 μm.

To investigate whether altered Wnt5A expression was associated with clinical chemoresistance in breast cancer patients, tumor biopsies obtained in a cohort of 24 patients during primary surgery naive to chemotherapy and secondary surgery after the tumor had recurred and after chemotherapy were analyzed. The patients all showed relapses after chemotherapy (Supplementary Table 1). Paired pre- and post-treatment samples (after one or several cycles of chemotherapy) were compared for Wnt5A and ABCB1 protein expression by immunohistochemistry staining (Supplementary Figure 4). Among the relapsed patients, 19 of the 24 (79%, *P*<0.001) of the biopsied samples showed significant increases of Wnt5A expression (Figures 8A, 8D & 8G), and 21/24 (88%, *P*<0.001) of the tumor biopsies also showed a significant upregulated ABCB1 expression after chemotherapy (Figures 8B, 8E & 8H). Furthermore, 18/24 (75%) of the samples showed significantly paired increases of Wnt5A and ABCB1 expression after chemotherapy. Only three tumor biopsies showed increased ABCB1 expression without concurrent increases in Wnt5A after chemotherapy. These data indicated high correlation between Wnt5A and ABCB1 in the clinical samples. Prompted by the observation of angiogenesis in the MES-SA/Dx5 xenograft model described above, the expression levels of VEGF were further investigated in the tumor biopsies from the relapsed patients. The data showed that among the 24 relapsed patients, 21 (88%, *P*<0.001) samples showed significant increases in VEGF expression (Figures 8C, 8F & 8I). Furthermore, 19/24 (79%) of the patient samples showed significantly paired increases of VEGF and ABCB1 expression after chemotherapy. Moreover, 17/24 (71%, *P*<0.001) of the samples showed significantly concurrent increases of VEGF and Wnt5A expression after chemotherapy. These data indicated positive correlation between Wnt5A, ABCB1 and VEGF in relapsed tumors, concurring with the observation of angiogenesis of the drug-resistant cell line xenograft model. Taken together, the relapsed tumors showed significant increases of Wnt5A or ABCB1 expression after chemotherapy. In spite of the relative small number of paired breast tumor samples analyzed, the results supports that Wnt5A activation is involved in chemoresistance of breast cancer, consistent of the *in vitro* data.

## DISCUSSION

### Interplay between the Wnt pathway and multidrug resistance

In recent years, several laboratories have started to focus on the relationship between the Wnt signaling pathway and multidrug resistance. Enhancing β-catenin signaling by treatment with GSK3β inhibitors in the brain vasculature endothelial cells leads to elevated ABCB1 expression [26]. The Wnt receptor, *Fzd1*, was upregulated in a doxorubicin-resistant neuroblastoma cell line [27], and *Fzd1*-slienced drug-resistant cells had reduced ABCB1 expression levels and were sensitive to chemotherapeutic treatment [19, 27, 28]. Colon cancer patients with mutant β-catenin had higher ABCB1 expression [29], and *Wnt* signaling was activated in a chemotherapy-resistant side-population (SP) of colon cancer cells. Human *ABCB1* expression level was monitored by several transcription factors including AP-1, EGR1, C/EBP, NF-κB and TCF4/β-catenin [30]. A number of TCF4/β-catenin binding sites are found on the ABCB1 promoter [30] which indicates that Wnt pathway-related proteins are dominant transcription factors in ABCB1 expression. Inhibition of β-catenin sensitized the cells to paclitaxel and irinotecan [31] in colon cancer SP cells and 5-FU in 5-FU-resistant cholangiocarcinoma [32]. ABCB1, which is known to be responsible for drug resistance in chronic myeloid leukemia (CML), decreases in expression levels after treating with β-catenin and Wnt1 shRNA [18]. Furthermore, induced Wnt/β-catenin signals by several proteins lead to increased *ABCB1* expression level and promote drug resistance in different cancer cells such as the PITX2 protein in renal cancer cell lines [33] and retinoic acid receptor gamma in cholangiocarcinoma [34]. These evidences support the observation presented in this work on upregulation of the Wnt/β-catenin pathway in drug-resistant MES-SA/Dx5 and MCF7/ADR2 cells. Our data indicated that the β-catenin-related pathway was upregulated in the drug-resistant MES-SA/Dx5 and MCF7/ADR2 cells.

### Role of the Wnt5A pathway in drug-resistance cancer

In a previous study, evidences indicated correlation between *Wnt5A* and cancer drug resistance. Upregulation of *Wnt5A* was found in oxaliplatin-resistant ovarian carcinoma cell line [35]. Ovarian cancer that overexpressed Wnt5A also showed lower chemosensitivity to paclitaxel, oxaliplatin, 5-fluorouracil, epirubicin and etoposide [36]. Furthermore, Wnt5A and its receptor, ROR2, were found to upregulate AKT/PKB survival signaling in histone deacetylase inhibitor-resistant colon cancer [37]. Wnt5A and NFATc2 were also upregulated in expression in pancreatic cancer; inhibition of *Wnt5A* led to significant increases in drug induced apoptosis both *in vitro* and *in vivo* [38]. In this study, higher Wnt5A expression observed in MES-SA/Dx5 and MCF7/ADR2 cells resulted in β-catenin activation by a noncanonical PKA pathway. Based on these evidences, Wnt5A is an important signal protein associated with drug-resistance in cancer.

### Relationship between the PKA pathway in drug-resistant cancer

Type 1 cAMP-dependent Protein Kinase A-related pathway is one of the pathways that modulates ABCB1 expression in multidrug-resistant breast cancer cells [39]. Selective PKA inhibitors were options to inhibit survival of drug-resistant cancer cells [40]. Abnormal activation of PKA increases DNA repair in cells which in turn results in resistance to DNA damage [41]. Moreover, activation of PKA leads to conversion of ER alpha inhibitor, tamoxifen, into a growth stimulator in tamoxifen-resistant breast cancer cells [42]. Hence, tamoxifen might become beneficial to breast cancer patients who have lower levels of PKA and ER alpha S305-P [43]. Elevated PKA signals were also observed in trastuzumab resistance in Her2-positive breast cancer [44]. PKA- and NF-kappa B-related signals are upregulated in castrate-resistant prostate cancer cells and a combined regime of inhibitors of PKA and NF-kappa B with docetaxel significantly induces apoptosis in castrate-resistant prostate cancer cells [45]. Furthermore, PKA-related genes are reported to be associated with MEK inhibitor resistance in colorectal cancer and 20-30% non-small cell lung cancer [46]. In this work, we observed that PKA regulated the β-catenin pathway in MDR cancer cells. Our data support the notion that PKA plays an important role in drug resistance.

### Methylation induced by doxorubicin in MDR cells

In our research, hypomethylation of the *Wnt5A* intron was found in drug-resistant MES-SA/Dx5 and MCF7/ADR2 cells and this methylation site regulated Wnt5A expression level in both cells. Based on the reported results, DNA methylation inhibitors, hydralazine and magnesium valproate, were combined with doxorubicin and cyclophosphamide used in the clinical breast cancer patients [47]. In addition, the global epigenetic changes played an important role in acquired drug-resistant phenotype of MCF7 drug resistance cells [48]. On the other hand, previous study indicated that DNA methylation status was affluence by chemotherapy drug in cancer cell and alternation of DNA methylation pattern led to gene expression level change such as *ABCB1* was hypermethylated in MCF7 compared to MCF7/ADR2 cell [49]. Hypomethylation genes, such as ABCB1, GSTpi, MGMT, Upa and TGM2 had lower methylation ability in MCF-7 doxorubicin-resistant cells and hypomethylation of ABCB1 led to higher ABCB1 expression [50, 51]. Demethylation of ABCG2 was observed and negatively associated with ABCG2 protein expression in mitoxantrone-resistant multiple myeloma cells [52]. Our finding provided another evidences that upregulation of Wnt5A controlled by DNA hypomethylation led to increases in the expression levels of β-catenin-regulated genes in MES-SA/Dx5 and MCF7/ADR2 cells.

### Correlation and clinical outcomes of Wnt5A and ABCB1 in clinical chemoresistance in breast cancer patients

Overexpression of ABCB1 in tumor tissues of cancer patients following chemotherapy is well established [53]. On the other hand, expression of FZD1 is highly correlated to ABCB1 expression level [27, 28]. Since there are only limited number of ABCB1 inhibitors currently available, continued searches for activated molecular targets in chemoresistant cancer are crucial for the development of novel strategies for treating chemoresistant patients [54]. Towards this goal, expression of Wnt5A and ABCB1 in biopsies from 24 pre-and post-chemotherapeutic-treated breast cancer patients were analyzed. The cohort included 24 patients with relapses most likely due to chemoresistance. Enhanced Wnt5A was detected in the tumour biopsies, and significantly elevated Wnt5A and/or ABCB1 expression was shown in biopsied samples after chemotherapy (Figures 8A & 8B). It is anticipated that a more extensive cohort of patients would confirm our observations, which are highly consistent with the *in vitro* data that Wnt5A and ABCB1 are both involved in chemoresistance and cell survival.

### Relationship between VEGF, Wnt5A and ABCB1 in multidrug-resistant cancer cells: a proposed mechanism

Higher expression levels of both secreted Wnt5A and secreted VEGF in multidrug-resistant MES-SA/Dx5, KB-V1, NCI/ADR-RES and SW620-MDR1 cells (Supplementary Figure 2). Furthermore, In the Wnt5A-knockdown xenograft model, decreased angiogenesis and reduced VEGF and ABCB1 expression levels were observed (Figure 6E). VEGF and ABCB1 are both β-catenin-targeted genes [18, 55]. In the previous study, increased Wnt5A, β-catenin and VEGF-A were found in non-small-cell lung cancer (NSCLC) [56], and Wnt5A expression was correlated to expression of VEGF, Ki67, androgen receptor in prostate cancer [57]. Despite the need to clarify the relationship between ABCB1 and VEGF, upregulation of VEGF-A and VEGF-B was found in NSCLC side population (SP) cells, accompanied by overexpression of ABCG2 and ABCB1 mRNAs [58]. Besides, ABCB1 and VEGF enhanced the invasion properties of MDR cells [59]. These are evidences to indicate that VEGF-related signaling is upregulated in MDR cancer cells. Based on our observation, VEGF inhibitor may be an option for inhibiting MDR cells.

**Figure 9:**
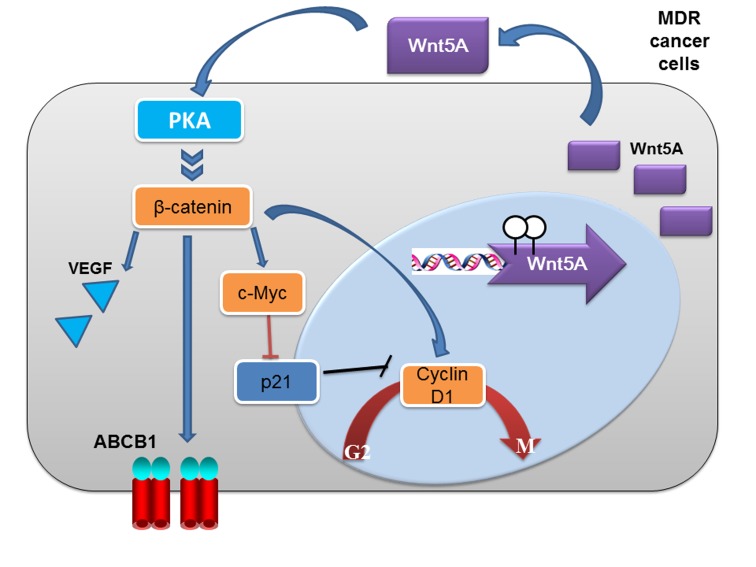
A model of Wnt5A/PKA/β-catenin activation in the development of drug resistance in cancer cells In the model, hypomethylation of Wnt5A probably leads to upregulated expression of Wnt5A in MCF7/ADR2 and MES-SA/Dx5 cells. Subsequently, Wnt5A activates PKA and PKA phosphorylates and inactivates GSK3β leading to activation of β-catenin and β-catenin-targeted genes including ABCB1, cyclin D1 and c-Myc. The upregulation of cyclin D1 and c-Myc led to increase cell proliferation and anti-apoptosis in MCF7/ADR2 and MES-SA/Dx5 cells under chemotherapy treatment. The proposed model, based on experimental evidences presented here, suggests that Wnt5A-mediated activation of downstream signal transducers is an important mechanism in the development of drug resistance in MCF7/ADR2 and MES-SA/Dx5 cells.

## CONCLUSIONS

This study presented results on the involvement of Wnt5A and the subsequent activation of the non-canonical PKA/β-catenin pathway in chemoresistance in two drug-resistant cell lines and in biopsied samples from breast cancer patients. Interestingly, VEGF upregulation was found in MDR cancers. These observations are useful for searching for novel therapeutic targets in chemoresistant patients.

## MATERIAL AND METHODS

### Cell culture

The multiple drug-resistant cell line MES-SA/Dx5-C5 (CRL-1977; ATCC, Manassas, VA) was established from the human sarcoma cell line MES-SA (CRL-1976; ATCC) in the presence of increasing doxorubicin concentrations [60]. Both cell lines were continuously maintained in McCoy's 5A medium supplemented with 10% fetal bovine serum and 2 mM antimycotics (Invitrogen Corp., Carlsbad, CA). MCF-7/ADR2 was established from the human mammary gland adenocarcinoma cell line MCF-7 (ATCC HTB22) in the presence of increasing doxorubicin concentrations followed by previous described [61]. Both cell lines were grown in Dulbecco's modified Eagle's medium supplemented with 10% fetal bovine serum and 2 mM antimycotics (Invitrogen). KB-V1 is a multidrug-resistant subclone derived from KB-3-1 (DSM ACC 158) [62]. Protein lysates of both cell lines were kindly provided by Dr. Chung-Pu Wu at Chang Gung University. NCI/ADR-RES is a multidrug-resistant subclone derived from OVCAR-8, an ovary carcinoma cell line [63]. Both the parental and drug-resistant cell lines were grown in Dulbecco's modified Eagle's medium supplemented with 10% fetal bovine serum and 2 mM antimycotics (Invitrogen). SW620-MDR1 is a multidrug-resistant subclone derived from SW620, a colon cancer cell line [64]. Both cell lines were kindly provided by Dr. Chuck C-K Chao at Chang Gung University and grown in Roswell Park Memorial Institute (RPMI) 1640 medium supplemented with 10% fetal bovine serum and 2 mM antimycotics (Invitrogen).

### Western blot analysis

Western blot analysis of indicated proteins was performed as described previously [65]. The antibodies used were as follows: anti-Wnt5A (sc-30224, Santa Cruz Biotechnology, CA), anti- MDR-1(sc-13131, Santa Cruz Biotechnology), anti-cMyc (sc-70496, Santa Cruz Biotechnology), anti- cyclin D1(sc-8396, Santa Cruz Biotechnology), anti-p-β-catenin, anti-p-GSK3β (sc11757, Santa Cruz Biotechnology, CA), anti-GSK3β (sc-9166, Santa Cruz Biotechnology), anti-β-catenin (13-8400, Invitrogen), and anti-β-actin (MAB1501, Millipore, Billerica, MA).

### H-89 and 5-aza-cytidine treatment

Each cell line was treated with H-89 (Santa Cruz Biotechnology, CA) at the indicated concentration for 48 h. Then, protein expression levels were assessed by western blot analysis. MES-SA cell line was treated with 5μM *5-aza*-cytidine (Sigma). Then, Wnt5A mRNA expression was assayed by quantitative PCR. DNA methylation status was assayed by COBRA assay.

### shRNA and plasmid construction

The Wnt5A shRNA clone (TRCN0000062717; CGTGGACCAGTTTGTGTGCAA) targeting at the human Wnt5A was purchased from the National RNAi Core Facility (Academia Sinica, Taipei, Taiwan). For construction of the red fluroscence protein (RFP) shRNA expression plasmids, the Blasticidin GSGF2A turboRFP fragment was obtained by AvrII and BamH I double digestion of TRC009 (Academia Sinica) and ligated to the AvrII- and BamH I-digested TRC001-based shRNA vector of clone TRCN0000062717. The resulting construct was designated as pRFP- shWnt5A.

### cAMP detection

Cells were plated at a density of 1 ×10^5^ cells/well in a 24-well plate. The cells were then treated with the indicated concentrations of PKA inhibitor, H-89. After 48 h of drug treatment, cells were assayed by cAMP Direct Immunoassay Kit, Colorimetric (Calbiochem) according to the manufacturer's instructions.

### Transient transfection

Cells were transfected with DNA of indicated concentration premixed with Lipofectamine 2000 (Invitrogen) as described previously [66]. After 48 h of incubation, cell medium was collect for detection of CRE activities by SEAP assays, and cells were lysed by the Passive Lysis Buffer (Promega) to perform TOP or ABCB1 transactivation activity detection by luciferase assays. Cell viability was determined in MTT assays and cells were assayed for cell cycle changes by PI staining. To determine apoptosis, TUNEL assays were performed.

### Antibody treatment

Cells were plated at a density of 1 ×10^5^ cells/well in a 24-well plate. The cells were then treated with the indicated concentrations of Wnt5A antibody (sc-30224, Santa Cruz) [67]. After 48 h of antibody treatment, cell lysates was collect for western blot, cell medium was collect for detection of CRE activities by SEAP assays, cells were lysed by the Passive Lysis Buffer (Promega) to perform TOP activity detection by luciferase assays and cells were collect for detection of drug pump-out ability by Calcein AM assay. After 24 h of antibody treatment, the cells were then treated with the indicated concentrations of doxorubicin. After 48 h of incubation,cell viability was determined in MTT assays.

### Combined Bisulfite Restriction Analysis (COBRA)

Genomic DNAs extraction and bisulfite conversion were performed as described previously [68]. Briefly, for the amplification of the human Wnt5A intron I, PCR was performed using 10 ng of the bisulfite-converted genomic DNA as a template. The primer sets of COBRA-PCR were listed as follows: The Wnt 5A COBRA 1 F sequence was 5′-TGTTTGAGTTTATTTAGTGG-3′. The Wnt 5A COBRA 1R sequence was 5′-AAAAAAACTAAAAACCAACTACTCC-3′. Purified PCR products were then digested with 10 U of Hha I (New England Biolabs) at 37°C. The quantification criterion of COBRA was as previously published [69].

### Quantitative real-time RT-PCR

The RT-PCR was performed as previously described [70]. Brief, total RNA (2 μg) was prepared from the transfectants and was treated with DNase I. RNA was reverse transcribed into cDNAs at 42 ^o^C for 60 min using Moloney Murine Leukemia Virus Reverse Transcriptase (Invitrogen). After the oligo(dT)-primed reverse transcription reaction, real-time PCR was performed in a LightCycler 480 (Roche, Mannheim, Germany) in 96-well plates. The reaction mixture was 1 μl RT product, 15 μl RealQ-PCR Master Mix Kit (Ampliqon AqS, Denmark), 1 μl each of 10 μM forward and reverse primers (Supplementary Table 2), and the total volume was adjusted to 10 μl with nuclease-free water. The real-time PCR program was: pre-incubation at 50 ^o^C for 2 min, initial denaturation at 95 ^o^C for 7 min, and 45 cycles at 95 ^o^C for 10 s, 60 ^o^C for 15 s and 72 ^o^C for 30 s. The program was terminated by a final extension at 60 ^o^C for 1 min and cooling at 40 ^o^C for 5 min. For normalization, the mRNA level of the β-actin gene in each RNA preparation was determined. Relative gene expression was determined by the ΔΔC_t_ method, where C_t_=threshold cycle. The relative targeted mRNA levels were normalized to the mRNA level of the reference β-actin gene. The melting curve of the amplification product was always checked to ensure a single clean peak that represented good-quality real-time PCR data.

### Cell viability MTT assay

Cells were plated at a density of 1×10^5^ cells/well in a 24-well plate. The cells were then treated with the indicated concentrations of various drugs or transfected with the indicated plasmid. After 48 h of incubation, the medium was removed and phosphate buffered saline (PBS) was used to wash the cells. Thiazolyl blue tetrazolium (Sigma-Aldrich) (200 μl) was added to each well and was incubated with the cells at 37 °C for 2 h. Subsequence, 400 μl DMSO was added to each well and incubation at 37 °C was continued for 20 min. Absorbance of the mixture was read at 540 nm using a Microplate Reader (VersaMax, Molecular Devices, Sunnyvale, CA). Cell viability (%) was calculated as the fraction of the surviving cells in each drug-treated experiment set to that of the control.

### Cell cycle analysis by FLOW cytometry

Cells were plated at a density of 1×10^5^ cells/well in a 24-well plate. After 24 h, the cells were transfected with the indicated plasmid or treated with the indicated antibody for 48 h. The cells were then harvested and fixed with 1 ml of 70% ice-cold ethanol. The fixed cells were pelleted and suspended in lysis buffer (0.5% Triton X-100 and RNAse A at 10 μg/ml) (Sigma-A) at 37 °C for 1 h. The cells were stained with 50 μg/ml propidium iodine (Chemicon). After 20 min of incubation at 4 °C in the dark, the cells were analyzed using a FCM scan flow cytometer using the CellQuest software (Becton Dickinson, San Jose, CA). The percentage of cells in the apoptotic sub-G1 and G1, S-phase and G2-M phase were calculated using the Modfit software (Verity Software House, Topsham, ME).

### TUNEL assay of cell death

Cell cultures were fixed and detection of DNA-strand breaks was performed by the terminal deoxynucleotidyltransferase-mediated UTP end labeling (TUNEL) method using the Apo-BrdU In Situ DNA Fragmentation Assay Kit (BioVision, Mountain View, CA) according to the manufacturer's instructions and as previous reported [71]. TUNEL staining-positive cells were detected by flow cytometry using Cellquest Pro FACS (BD Biosciences, San Jose, CA).

### Calcein-acetoxymethyl ester assays for ABCB1 functions

A calcein acetoxymethylester (calcein AM) assay was performed using the MultiDrugQuantTM Assay kit (CHEMICON). Briefly, cells were plated at a density of 1×10^5^ cells/well in a 24-well plate. After 24 h, the cells were transfected with the indicated plasmid for 48 h. Calcein-AM was added at 25 nM into each well and the cells were incubated for 20 min before being trypsinized with 0.25% trypsin-EDTA for 2 min and pelleted. Supernatant was removed after centrifugation at 800 *g*. One ml medium was used to resuspend the cell pellet for FLOW cytometry analysis using Cellquest Pro FACS (BD Biosciences, San Jose, CA, USA). Distribution of the green-fluorescent protein calcein in the cells was observed by FLOW cytometry. The percentage relative fluorescence (RFL) in the cells was expressed as: % FL = [(FL treatment - FL nontreatment)/FL nontreatment] × 100%.

### Tumor xenografts in nude mice

Eight week-old male nude mice were purchased from the National Laboratory Animal Center (Taipei, Taiwan). All experimental procedures involving the use of animals for experimental purposes were approved by the Institutional Animal Care and Use Committees (IACUC) of Chang Gung University under the protocol number CGU11-113. The mice were maintained in an air-conditioned animal facility under constant temperature and humidity conditions with a 12:12 light-dark cycle and the mice were allowed *ad libitum* in the diet or drinking water. Mice were randomly picked to different groups and each group had 5 or more mice. Wnt5A shRNA or control scramble shRNA-transfected MES-SA/Dx5 cells (1×10^6^) was mixed with Matrigel (BD Bioscience, San Jose, CA) followed by subcutaneous injection into the dorsal region near the thigh of the mice. Upon 1 month of tumor establishment, mice were intraperitoneally injected with the indicated concentrations of doxorubicin or PBS. The tumor volume and IVIS image were obtained on day 0, 7 and 14 after doxorubicin treatment. The tumor volume (V) was measured by a vernier scale every 7 days up to 3 weeks, and calculated as V (mm^3^)=(L×W^2^)/2, where L was the largest diameter and W was the diameter perpendicular to L. On day 21 of doxorubicin treatment, all mice were sacrificed and tumors were resected, weighed and frozen or fixed in formalin and paraffin embedded for immunohistochemical studies.

### Bioluminescent IVIS imaging System

Xenografted nude mice were kept in a chamber and were anesthetized with 2% isofluorane/oxygen mixture and intraperitoneally injected with 20 mg/ml D-luciferin (Promega) in PBS (200 mg/kg). Mice were sedated using isoflurane, and live anesthetized mice were imaged using the Bioluminescent IVIS Imaging system (Xenogen Corp., Alameda, CA). The resulting light emission was quantified using the Living Image software (Xenogen). Raw values were reported as photons/second/cm^2^/sr.

### Breast cancer patients and immunohistochemical staining studies

The 24 tumor paired tumor tissue biopsied samples for immunohistochemical analyses were obtained from breast cancer patients at diagnosis and after treatment from 1995 to 2014 at Chang Gung Memorial Hospital (CGMH)-Linkou Medical Center. Tumor materials were collected after informed consent and with approval from Institutional Review Board of CGMH (IRB:101-4592B). All patients received surgery and treatment according to international protocols, including doxorubicin-based chemotherapy. Biopsies of paired cancer samples were obtained from the patients immediately after surgery at diagnosis, and after at least one course of chemotherapy. Immunostaining was performed with anti-ABCB1, anti-VEGF and anti-Wnt5A antibodies (Santa Cruz) using the Dako-REAL, Alkaline-Phosphatase/RED detection system (Dako, Glostrup, Denmark). Hematoxylin was used for counterstaining according to the manufacturer's protocol. The staining intensity of each malignant tissue was evaluated and scored by pathologists. The Wnt5A, ABCB1 and VEGF-A staining was scored for a tumor using an H-score that had been obtained by multiplying the staining intensity (graded as 0, negative; 1+, weak; 2+, moderate; and 3+, strong) (see Supplementary Figure 3) by the percentage of tumor cells with positive cytoplasmic staining (0% to 100%). H-score to verify the IHC results was as described previously [72, 73].

### Statistical analysis

Statistical analyses were performed using GraphPad Prism 5 (GraphPad Software). The surviving fraction and the relative luminescence unit (RLU) were measured in triplicate samples and were expressed as mean ± S.D. The Student's *t*-test was used for statistical analysis. Immunohostochemical results (staining intensity × percentage) of Wnt5A, ABCB1 and VEGF immunoreactivity in human breast cancer biopsies were assessed using the Wilcoxon test. *P*<0.05 was considered as statistically significant.

## SUPPLEMENTARY MATERIAL, FIGURES AND TABLES


