# Supplementary material for: Wnt5A regulates ABCB1 expression in multidrug-resistant cancer cells through activation of the non-canonical PKA/β-catenin pathway
Source: Oncotarget. 2014 Oct 24;5(23):12273–90. doi: 10.18632/oncotarget.2631 (PMC4322984; doi:10.18632/oncotarget.2631)
Supplement: Supplementary file 1 [file oncotarget-05-12273-s001.pdf]

Wnt5A regulates ABCB1 expression in multidrug-resistant cancer cells through activation of the non-canonical PKA/β-catenin pathway

Supplementary Material

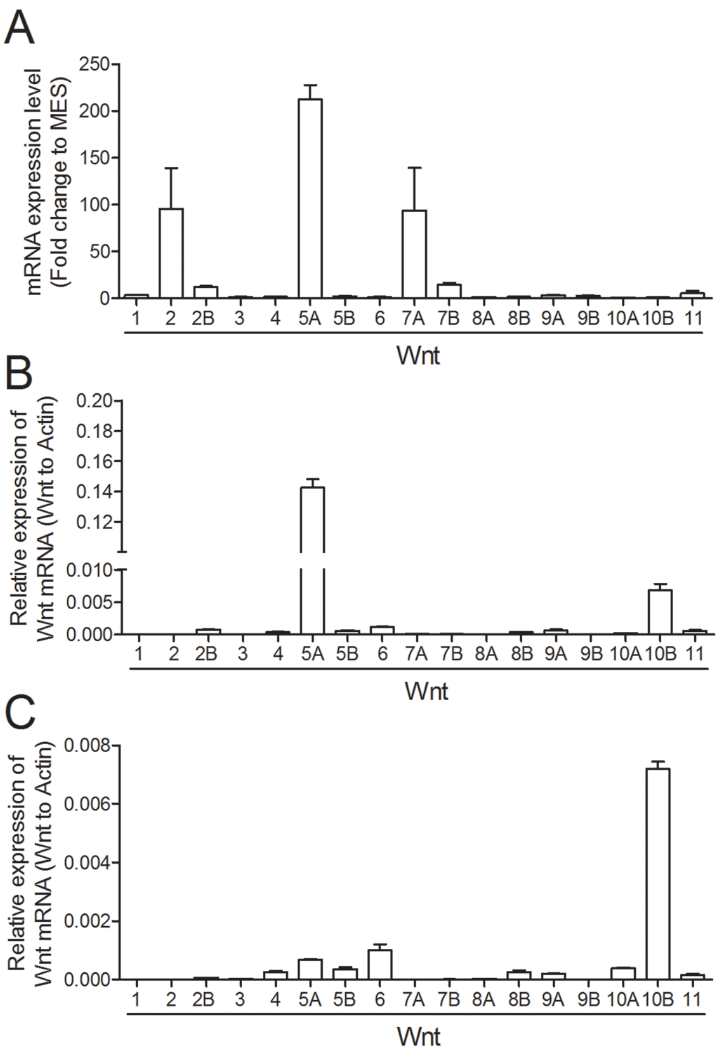

Supplementary Figure 1: mRNA expression levels of Wnt's in two MDR cell lines as determined by real-time RT-PCR. (A) Expression ratio Wnt's of MES-SA/Dx5 relative to MES-SA. (B-C) mRNA expression levels of Wnt's in MES-SA (B) and MES-SA/Dx5(C) cells.

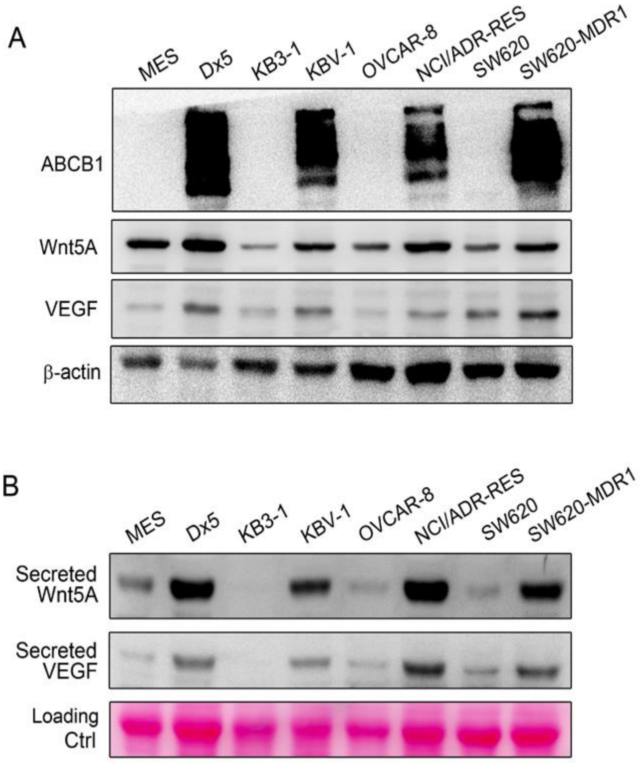

Supplementary Figure 2: The ABCB1, Wnt5A and VEGF are upregulated in drug-resistant cancer cells. Western blot analysis of (A) ABCB1, Wnt5A and VEGF and (B) secreted Wnt5A and VEGF protein expression levels in four pairs of parental and multidrug-resistance cancer cell lines, MES-SA, MES-SA/Dx5, KB3-1, KBV-1, OVCAR-8, NCI/ADR-RES, SW620 and SW620-MDR1 cells. Equal protein loading in each lane was confirmed by Ponceau S staining of the PAGE gels.

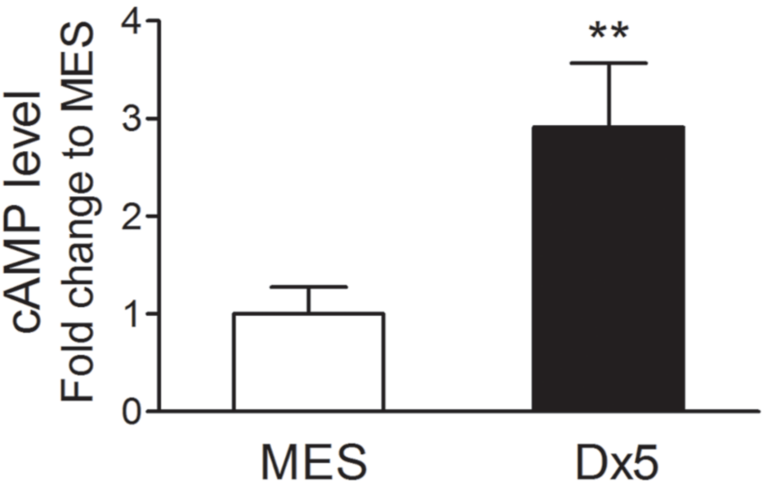

Supplementary Figure 3: Cyclic AMP levels in MES-SA/Dx5 cells. The cAMP level in MES-SA and MES-SA/Dx5 cell line were assayed by cAMP Direct Immunoassay Kit. Data represent means ± S.D. of three independent experiments. *P<0.05 and **P<0.01 indicate differences between the drug-resistant and the parental cells.

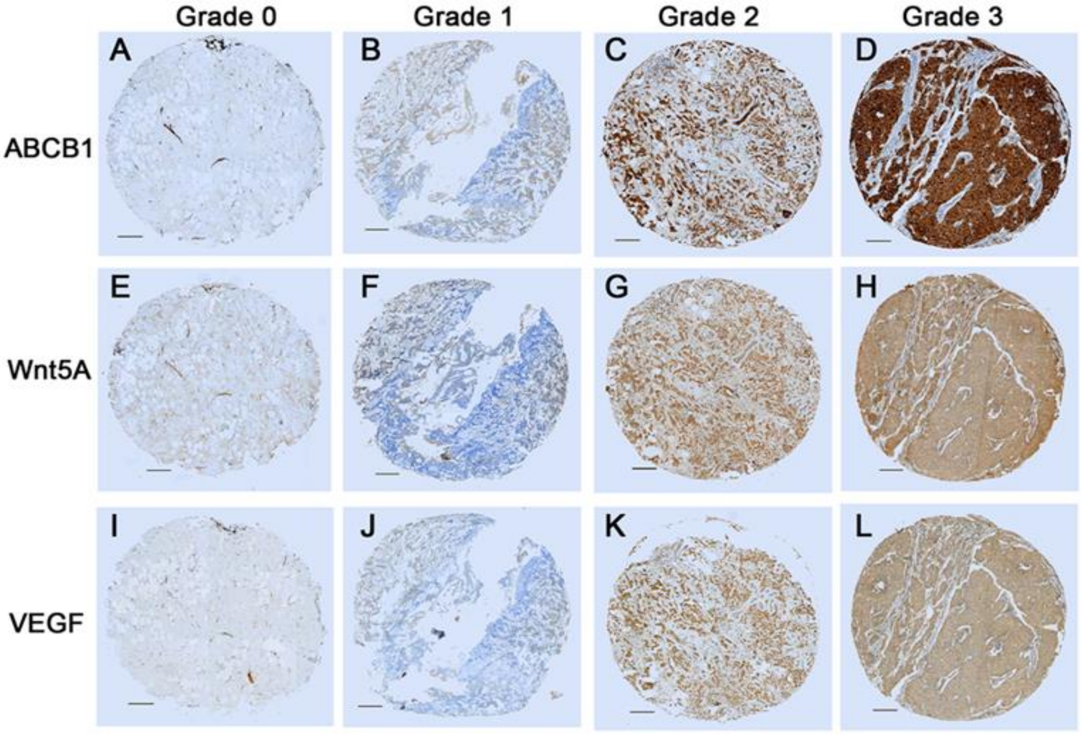

Supplementary Figure 4: ABCB1, Wnt5A and VEGF expression in human breast cancer. Expression of ABCB1, Wnt5A and VEGF in breast cancer tissue microarrays (TMAs). No immunoreactivity is seen in non-specific negative controls for CD44v3-10 (A), MDR1 (E) and MCT4 (I). Representative images shown are of Grade 1 (weak) ABCB1 (B), Wnt5A (F) and VEGF (J); Grade 2 (medium) ABCB1(C), Wnt5A(G) and VEGF(K); and Grade 3 (strong) ABCB1 (D), Wnt5A(H) and VEGF(L) immunostaining. Scale bar inserts indicate the typical areas of staining at high amplification (200 X).

Supplementary table 1: Clinical characteristics of patients and their respective Wnt5A and ABCB1 expression level

Patient	Age of diagnosis	Pathologic Characteristics	PR	ER	HER2	Date of death	pre-treatment ABCB1 expression (IRS)	pre-treatment Wnt5A expression (IRS)	pre-treatment VEGF expression (IRS)	post-treatment ABCB1 expression (IRS)	post-treatment Wnt5A expression (IRS)	post-treatment VEGF expression (IRS)
1	41	IDC	U	U	P		90	150	180	90	120	300
2	48	IDC	N	N	N	2007/2/28	60	140	140	270	200	300
3	35	IDC	P	P	N	2011/4/19	100	130	120	270	270	300
4	44	IDC	N	P	P		150	150	100	180	180	180
5	55	IDC	P	P	P		50	0	30	270	200	250
6	55	IDC	P	P	N	2009/12/1	30	60	50	300	300	150
7	52	IDC	U	U	N	2010/10/99	30	60	120	120	150	240
8	42	IDC	N	N	N		80	150	135	300	300	270
9	34	IDC	P	P	P		100	80	120	270	270	260
10	50	LCIS	P	P	P	2004/12/2	120	50	90	240	150	200
11	36	IDC	U	U	N	2002/5/10	90	60	80	100	100	200
12	52	LCIS	U	U	N		150	100	150	300	210	160
13	52	IDC	P	P	P	2006/10/21	40	60	125	300	210	300
14	51	IDC	P	P	P	2008/8/8	40	150	130	160	160	270
15	51	IDC	P	-	P		180	160	160	270	270	270
16	24	IDC	P	P	P		140	160	160	300	300	300
17	39	IDC	P	P	P		40	50	50	240	240	240
18	49	IDC	P	P	P		180	140	160	300	180	300
19	34	IDC	N	N	P		180	140	140	270	240	240
20	37	IDC	P	P	P	2012/9/5	90	100	120	240	240	240
21	62	IDC	P	P	P	2011/11/15	100	50	100	210	200	180
22	57	IDC	N	P	P		90	80	270	240	160	180
23	53	IDC	N	N	P		90	80	40	300	200	300
24	47	IDC	N	N	P		100	40	40	300	300	300

IDC: Infiltrating duct carcinoma

LCIS: Lobular carcinoma in situ

P: positive

N: negative

U: unknown

Supplementary table 2: List of primers used for amplification using Quantitative RT-PCR.

Gene Name	Forward Primer (FP) or Reverse Primer (RP)
Wnt1	FP: 5’-TTCCTCATGAACCTTCACAACA-3’
	RP: 5’-GCGATTTCTCGAAGTAGACGAG-3’
Wnt2	FP: 5’-GTGATCCAAAGAAGATGGGAAG-3’
	RP: 5’-TACACGAGGTCATTTTTCGTTG-3’
Wnt2B	FP:5’-ACAATATCCCTGGTTTGGTGAG-3’
	RP: 5’-CACCGTAGTGGATGTTGTCACT-3’
Wnt3	FP:5’-GCCTCTGACAAGCCCGAA A-3’
	RP:5’- GCGACGCCCCCAATAGTT-3’
Wnt3A	FP:5’-CTGTAGCGAGGACATCGAGTTT-3’
	RP:5’-GGGCACCTTGAAGTAGGTGTAG -3’
WNT4	FP:5’- CCT TCG TGT ACG CCA TCT CT-3’
	RP:5’- TCA GAG CAT CCT GAC CAC TG-3’
WNT5A	FP:5’-AGGGCTCCTACGAGAGTGCT-3’
	RP:5’-GACAC CCCATGGCACTTG-3’
WNT5B	FP:5’- TTTGGGAGAGTCATGCAGATAG-3’
	RP:5’- CGTCTGCCATCTTATACACAGC-3’
WNT6	FP:5’- GAGAGTGCCAGTTCCAGTTCC-3’
	RP:5’- TAAAGAGCCTCGACTTCTCGTC-3’
WNT7A	FP:5’-TGCCCGGACTCT CATGAA C-3’
	RP:5’- GTGTGGTCCAGCACGTCTTG-3’
WNT7B	FP:5’-TCTACGTGTTTCTCTGCTTTGG-3’
	RP:5’-GTTGTAGTAGCCCTGCTT CTCG-3’
WNT8A	FP:5’- ATGGGTCAAACAATGGAAAAAC-3’
	RP:5’- CCTGGTCATACTTGGCCTTTAG-3’
WNT8B	FP:5’- TAACTGTGGCTGTGATGACTCC-3’
	RP:5’- GTAGAGATGGAGCGAAAGGTGT-3’
WNT9B	FP:5’-CAACCTCAAGTACAGCACCAAG-3’
	RP:5’-GTGAG TCCTCCATGTACACCAG-3’
WNT10A	FP:5’-GAGGCACCGGAGTTGTCGC-3’
	RP:5’-TCCGACTCGGGTCAGGGCTC-3’
WNT10B	FP:5’- CCATCTTCATTGATACCCACAA-3’
	RP:5’- ACCCACTCTGTAACCTTGCACT-3’
WNT11	FP:5’- AGTTTTCCGATGCTCCTATGAA-3’
	RP:5’- CTTCATGCAGAAGTCAGGTGAG-3’
